# OLDER ADULT FALLS PREVENTION: THREE ADOPTED CLINICAL STRATEGIES FROM EVALUATION OF THE CDC STEADI INITIATIVE

**DOI:** 10.1093/geroni/igz038.2096

**Published:** 2019-11-08

**Authors:** Jennifer Edwards, Brittany Bickford, Yvonne Johnston, Aaron Alford

**Affiliations:** 1 National Network of Public Health Institutes, Washington, District of Columbia, United States; 2 National Network of Public Health Institures, New Orleans, Louisiana, United States; 3 Binghamton University, Binghamton, New York, United States

## Abstract

This evaluation examines patients’ barriers and facilitators to adopting an evidence-based fall prevention strategy. Twenty-one patients were telephone interviewed. The purposive sample includes patients over age 65, screened as at risk for falls, and who received a referral for falls risk intervention. Seven themes emerged from the qualitative analysis of interview transcripts: 1. Behavioral Facilitators, 2. Personal Fall Experiences, 3. Informed Decision-making, 4. Providers, 5. Friends and Family, 6. Home Setting Facilitators, and 7. Risk Perception. Three opportunities were identified: 1. Develop an outpatient follow-up protocol, 2. Develop a falls screening public service announcement, and 3. Partner with the local Office for Aging to connect patients at risk with community programs such as Tai Chi. A systems approach involving the CDC, National Network of Public Health Institutes (NNPHI), Broome County Health Department, and an Upstate New York hospital system’s outpatient practices was vital for the success of this evaluation.

